# The complete chloroplast genome of *Polygala japonica* Houtt. (Polygalaceae), a medicinal plant in China

**DOI:** 10.1080/23802359.2020.1861998

**Published:** 2021-01-21

**Authors:** Yang Zuo, Yanzhen Mao, Shuning Shang, Guo Yang

**Affiliations:** a School of Life Science, Shaoxing University, Shaoxing, China; b College of Forest, Nanjing Forestry University, Nanjing, China

**Keywords:** *Polygala japonica*, complete chloroplast genome, phylogenetic analysis

## Abstract

*Polygala japonica* Houtt. (Polygalaceae) is a perennial herbaceous plant widely distributed in south China. Here, we assembled and characterized the complete chloroplast (cp) genome of *P. japonica* using sequencing data. The complete cp genome of *P. japonica* was 165,439 bp in length, with a GC content of 36.7%. The complete cp genome shows a typical quadripartite structure with a pair of inverted repeats (IRs) of 36,786 bp, separated by a large single copy region (LSC) of 83,722 bp, and a small single copy region (SSC) of 8,145 bp. A total of 135 genes were annotated in the cp genome of *P. japonica*, consisting of 89 protein-coding genes, 8 rRNA genes, and 38 tRNA genes. The phylogenetic analysis showed *P. japonica* had a closer relationship with *P. tenuifolia.*

*Polygala japonica* Houtt. (Polygalaceae), locally called ‘gua zi jin’, is a perennial herbaceous plant widely distributed in south China. The whole plant has been used in folk medicine as an expectorant, anti-inflammatory, ataractic and antibacterial agents (Wang et al. 2008; Zhou et al. 2018). Many kinds of constituents have been found in this plant, such as flavones, saponins and steroids (Li et al. 2006). However, little is known about the genomic data of this medicinal plant. The plastome DNA marker sequences reported here could provide a valuable genetic resource for genetic diversity, phylogenetic evolution and taxonomy studies of the Polygalaceae family.

Plants of *P. japonica* were collected in Funiu Mountain (Henan, China; 112.5°E, 32.9°N). Plant specimens were deposited in the herbarium of Shaoxing University (accession number: SXU-20200415AM04). Genomic DNA was isolated by the modified method CTAB. After DNA isolation, 1 µg of purified DNA was fragmented and used to construct 350 bp short insert libraries, then sequenced with PE150bp on the BGISEQ500 sequencer (BGI, China). The filtered reads were assembled by the program SPAdes assembler 3.10.0 (Bankevich et al. 2012). Annotation was performed using the GeSeq (Tillich et al. 2017) and BLAST search.

The complete chloroplast (cp) genome of *P. japonica* (GenBank accession number: MT762167) was 165,439 bp in length, with a GC content of 36.7%. The complete cp genome shows a typical quadripartite structure with a pair of inverted repeats (IRs) of 36,786 bp, separated by a large single copy region (LSC) of 83,722 bp, and a small single copy region (SSC) of 8,145 bp. A total of 135 genes were annotated in the cp genome of *P. japonica*, consisting of 89 protein-coding genes, 8 rRNA genes, and 38 tRNA genes. 11 protein-coding genes, 7 tRNA genes, and 4 rRNA genes are duplicated in the IR regions. 22 genes contained two introns and 1 gene (ycf3) contained three introns. The maximum-likelihood method was used to infer the phylogenetic relationship. Phylogenetic analysis based on chloroplast genome sequences of eleven plants from Fabales shows that *P. japonica* is a sister to *P. tenuifolia* forming monophyletic group closely related to *P. fallax* and *P. arillata* (Figure 1), which is consistent with previous molecular results (Pastore et al. 2019).

**Figure 1.**
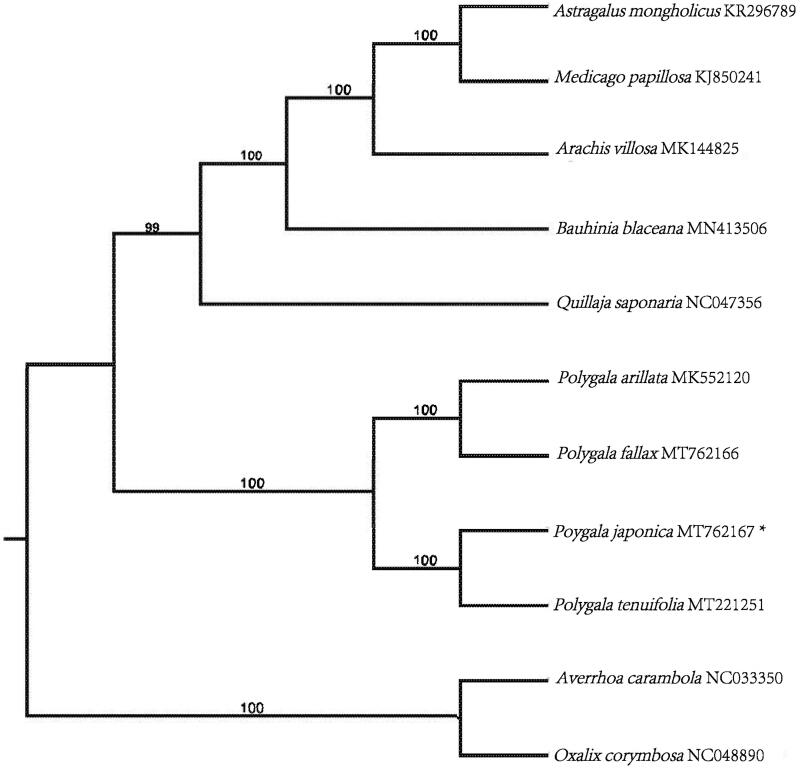
Maximum likelihood (ML) phylogenetic tree inferred from 11 complete cp genomes. The position of Polygala japonica is marked with an asterisk. Averrhoa carambola and Oxalis corymbosa were selected as outgroup. Values along branches correspond to ML bootstrap percentages. Genbank accession numbers were shown..

## Data Availability

The genome sequence data that support the findings of this study are openly available in GenBank of NCBI at (https://www.ncbi.nlm.nih.gov/) under the accession no. MT762167.1. The associated **BioProject**, **SRA**, and **Bio-Sample** numbers are PRJNA681297, SRR13161062, and SAMN16948893 respectively.
